# Identifying priority conservation areas in a Saharan environment by highlighting the endangered Cuvier’s Gazelle as a flagship species

**DOI:** 10.1038/s41598-020-65188-6

**Published:** 2020-05-19

**Authors:** F. Javier Herrera-Sánchez, Jose María Gil-Sánchez, Begoña Álvarez, Inmaculada Cancio, Jesus de Lucas, Ángel Arredondo, Miguel Ángel Díaz-Portero, Javier Rodríguez-Siles, Juan Manuel Sáez, Joaquín Pérez, Emil McCain, Abdeljebbar Qninba, Teresa Abáigar

**Affiliations:** 1 Harmusch, Study and Conservation of Wildlife. C/San Antón 15, 1°, E 13580 Almodóvar del Campo, Ciudad Real Spain; 2 Département de Zoologie et Ecologie Animale. Institut Scientifique de Rabat, Université Mohammed V. Av. Ibn Battouta, BP 703, 10090 Agdal, Rabat, Morocco; 3 Estación Experimental de Zonas Áridas (CSIC), Crta. Sacramento s/n, 04120-La Cañada de S. Urbano, Almería, Spain

**Keywords:** Biogeography, Conservation biology, Zoology

## Abstract

Monitoring populations and designing effective conservation actions for endangered species present significant challenges. An accurate understanding of current distribution, ecological traits and habitat requirements is imperative in formulating conservation strategies. Recent surveys on the southernmost Cuvier’s Gazelle (*Gazella cuvieri*) population, an ungulate endemic to North Africa, showcase its importance in terms of numbers and genetic diversity. This population inhabits a remote region in the extreme north-western portion of the Sahara Desert and has not been well studied. Here, we examine the potential distribution of Cuvier’s Gazelle and the environmental factors limiting the species in a Saharan environment, by combining broad-scale field survey data and species distribution models. Our objective was to identify high priority conservation areas in the southernmost known portion of the species’ distribution by modelling habitat selection at the landscape scale using a predictive distribution map. Our results show that the distribution of Cuvier’s Gazelle is strongly related to mountainous areas with heterogeneous terrain and remoteness from large human settlements over other ecological factors that had less impact on the species’ presence and distribution. We also provide a quantitative estimate of the potential distribution range of Cuvier’s Gazelle in southern Morocco, identifying two well-demarcated key areas. The two core areas currently contain enough rugged terrain isolated from human encroachment to support the endangered species in this harsh desert environment. We encourage the implementation of conservation planning for Cuvier’s Gazelle as an “*umbrella species*”, which will confer effective protection to higher-quality habitat zones and co-occurring species, leading to sustainable and ecologically responsible development in the region.

## Introduction

Knowledge of the population distribution and dynamics of threatened species in the wild is key to effective conservation actions^1,2^. While this may seem obvious, at present, there are many examples of endangered wildlife for which their current situation is unknown, especially in remote areas of the world and in less developed^3^. Field research through large-scale surveys plays a key role in obtaining quality data on species status; however, unfortunately, empirical field studies have decreased appreciably in the past several decades^4,5^. A recent study has shown that the proportion of fieldwork-based investigations in the conservation literature has dropped significantly from the 1980s until today: fieldwork-based publications decreased by 20% in comparison to a 600% and 800% increase in synthetic modelling and data analysis studies, respectively^6^. As a direct result, key decisions establishing national and global priorities in biodiversity conservation are lacking observational data. Researchers, funders and journals have been urged to conduct, fund and disseminate relevant field research^6^.

The conservation status and distribution of the Sahara’s megafauna is a paradigmatic case to illustrate this lack of fundamental and key data for well-designed conservation actions in remote areas. Field studies are becoming increasingly necessary because of the collapse in Saharan wild ungulate and mammalian carnivore populations over the last century^7^. In this remote region of northern Africa, any field work faces great logistical and safety challenges, due to remoteness, lack of infrastructure, extreme environmental conditions and, in many cases, ongoing armed conflicts^8^. One of the species that inhabits this desert is the endangered Cuvier’s Gazelle (*Gazella cuvieri* Ogilby 1840), a medium-sized ungulate endemic to North Africa. Over the last century, the Cuvier’s Gazelle population has undergone major fragmentation and its numbers have declined dramatically to 2,360–4,560 individuals due to overhunting and habitat loss^9–13^. Currently, the distribution of Cuvier’s Gazelle is limited to the Atlas Mountains and the neighbouring mountain ranges in Morocco, Algeria and Tunisia^11,13–15^. It is included within the subset of Sahelo-Saharan antelopes along with dama gazelle (*Nanger dama*), dorcas gazelle (*Gazella dorcas*), slender-horned gazelle (*G. leptoceros*), scimitar-horned oryx (*Oryx dammah*) and addax (*Addax nasomaculatus*). These species are well-adapted to life in the extreme conditions of this region, such as high temperatures, drought and a seasonal lack of food^7,10^. Like other Sahelo-Saharan antelopes, Cuvier’s Gazelle is poorly studied due mainly to its elusiveness and remote habitats^7,16^. Therefore, the limited knowledge about its biology and actual conservation status may lead to a lack of protection and even local extinctions. Cuvier’s Gazelle is defined as “Endangered” in Morocco, Algeria and Tunisia^10^ but globally classified as “Vulnerable” to extinction^3^. Given its importance in North Africa, the IUCN has elaborated an international strategy that includes several actions focussed on protection from illegal hunting, management of habitat, monitoring and environmental sensibility to achieve better preservation and recovery of populations^13^. Only through increasing knowledge regarding its natural history and habitat requirements will it become possible to identify the areas that need protection for more effective conservation of the species.

Changes are currently being proposed that would reclassify Cuvier’s Gazelle as a mountain ecotype of slender-horned gazelle^17^, a species strictly associated with the great sand desert and ergs of the Sahara^18^. Covering a wide variety of habitats from sea level to 2600 metres, Cuvier’s Gazelle mainly lives in mountain ranges and associated plateaus, such as semi-arid open Mediterranean forests of cork oak (*Quercus suber*), holm oak (*Quercus ilex*), *Pinus* spp., sandarac (*Tetraclinis articulata*), Atlas cedar (*Cedrus atlantica*), argan (*Argania spinosa*) and Phoenicean juniper (*Juniperus phoenicea*), but also in steppes, maquis or scrubland areas, and even in cereal fields in Algeria and Morocco^9,11,14,15,19^. In contrast, Cuvier’s Gazelle appears to avoid areas covered in heavy snow at high altitudes in winter and ranges to the Sahara Desert on rocky mountains and desert plateaux, limited to areas with argan and thorn trees *Acacia* spp. forests^9^. In this environment, recent surveys in the Bas Drâa-Aydar region (Morocco) show promising distribution and numbers of Cuvier’s Gazelle^20^, since this population has been described as one of the largest populations of the species, with 935 individuals (95% CI 597–1607)^20^. This population of Cuvier’s Gazelle inhabits the extreme north-western portion of the Sahara Desert and is probably the most important in terms of numbers and genetic diversity, making it essential to the species’ longevity and conservation^20^. In this region, with apparently suboptimal conditions, the topography, food availability and extreme climatic conditions may be crucial factors determining the species’ presence, but human settlements may also play a role and may restrict Cuvier’s Gazelle presence and usable habitat^9^.

In this study, we applied species distribution models, also known as habitat suitability models, a technique that combines information on species occurrence or abundance with environmental estimates and/or spatial characteristics^21–23^. Species distribution models are mainly used to address the potential effects of climate change on species distribution^24–26^ but are also used to improve the understanding of ecological factors for conservation planning and to detect unknown potential distribution areas for rare species^27–29^. Our goal is to identify the main ecological features of the southernmost subset of the Cuvier’s Gazelle population in the Sahara; for this purpose, we integrated data from a broad-scale field survey carried out from 2011 to 2014 and species distribution models to model habitat selection at the landscape scale, using a predictive distribution map. The final purpose of this study is to contribute to the biological conservation management of Cuvier’s Gazelle by identifying the extent of its potential habitat and high priority areas to ensure the species’ survival in the southernmost area of its distribution.

## Methods

### Study area

**Figure 1:**
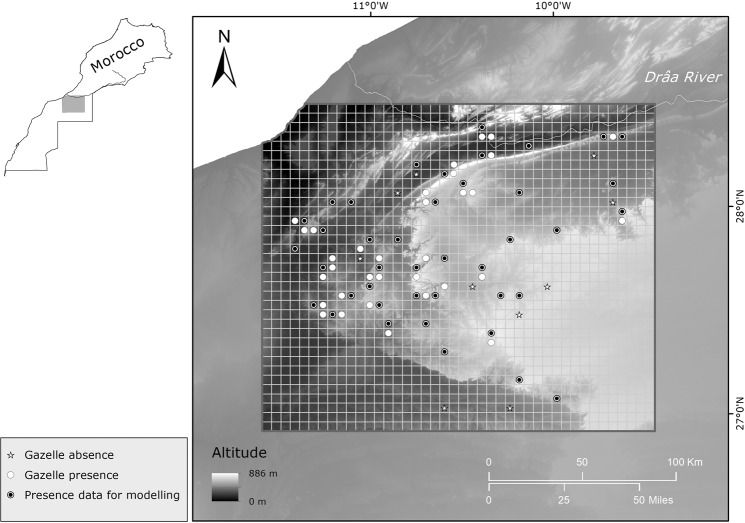
Location and topography of the study area in the north-western Sahara Desert, Morocco. Presence-absence data collected are shown in plots of 5 × 5 surveyed grids by *white dots* and *white stars*, respectively. *Black dots* show final presence data for building the species distribution model. Basemap image by Shuttle Radar Topography Mission (SRTM)^38^.

The survey was conducted in the extreme north-western part of the Sahara Desert between 28°30′–26°50′N and 11°40′–9°25′W in the Guelmim-Es Semara region (Fig. 1). The area is delimitated by two important geographical features: the lower Drâa River to the north and the upper basin of the Sequiat Al Hamra to the south. It is a typical Saharan landscape with a subtropical desert and a low-latitude hot, arid climate (Köppen-Geiger classification^30^). The mean, minimum and maximum temperatures are 22.7, 8.0 and 39.0 °C in the western zones (closer to the Atlantic Ocean), 23.2, 0.0 and 43.0 °C in the eastern zones, and 19.1, 10.7 and 29.0 °C in the northern zone. Total annual precipitation (with large interannual variability) is 138, 59 and 190 mm, respectively (recorded at climate stations at Smara, 26°46′N, 11°31′W; Tindouf, 27°40′N, 8°7′W; and Tan Tan, 28°26′N, 11° 06′W). The area contains diverse terrain with rough and hilly areas (*jbels*), flat areas with saline depressions (*sebjas*), plateaux (*hammadas*), clay plains (*dayas*), stony plains (*regs*) and some small dune areas (*ergs*). There are large mountainous reliefs: Aydar Mountain (western zone); three main *jbels*: Zini, Rich and Ouarkziz, the last with an approximate total length of 400 km (250 km in Morocco), and at the eastern edge, the Hammada of Tindouf. The whole region is a contact point between two ecoregions: the Sahelo-Arabian and the Macaronesian regions. Thus, is it possible to find features typical of Mediterranean, tropical vegetation and Macaronesia^31–33^. Woody vegetation is scarce and is mainly located in ravines and *oueds* (ephemeral sandy rivers) with open savannah-like forests of horn trees such as acacia (*Acacia raddiana*), sometimes along with balanites (*Balanites aegyptiaca*) and calotrope or sodom apple (*Callotropis procera*), and abundant African tamarisk (*Tamarix africana)* bushes along the gueltas. Other typical species are argan (an endemic tree of Morocco that reaches its southernmost limit here), *Periploca laevigata*, *Launaea arborescens*, sumac (*Rhus tripartitum*), *Maerua crassifolia* and *Euphorbia officinarum*, this latter being a key species from a more continental Macaronesian region. There is also important extremophilic vegetation that survives in the *hammadas*, *regs* and *ergs*, such as rose of Jerico (*Anastatica hierochuntic*), *Panicum turgidum*, *Nucularia perrinii*, colocynth (*Citrullus colocynthis*) and *Mesembryanthemum cryptanthum*.

### Field surveys and environmental predictor variables

Presence-absence data were collected from April 2011-April 2014 by completing walking surveys and sampling direct sightings and indirect signs, such as tracks, isolated dung piles and dung middens (accumulations of dung piles in latrines)^34–37^. After 194 transects on foot counting a total of 2169 km, 615 dung points were georeferenced and 61 gazelles sighted at 21 different sites. The mean distance covered by walking surveys was 12.08 ± SE 0.72 km (range 3.8–22.5 km). The correct identification of indirect signs was tested by genetic analyses at the Research Centre for Biodiversity and Genetic Resources at Porto University, Portugal, using methods described in Silva *et al*.^17^. For more detailed descriptions on the field data, see Gil-Sánchez *et al*.^20^.

To develop models that predict habitat suitability and determine the influence of variables on Cuvier’s Gazelle presence, we selected a set of previously published metric variables ranked in five categories (Table 1). Mean values for topographic morphology metrics (altitude, slope and topographic ruggedness index) and two temperature-moisture metrics (heat load index and compound topographic index) were obtained from Shuttle Radar Topography Mission (SRTM) with 1 arc-second (~30 m^2^) spatial resolution^38^. For topographic distance and human factor metrics, we used the Euclidean distance computed in ArcGIS 10.4^39^, measuring the distance to the coast and a two-tiered settlement size classification (DISTSETT1 and DISTSETT2). To describe vegetation, we used a primary productivity parameter: the average annual maximum green vegetation fraction or AMGVF^40^. AMGVF data are based on 12 years (2001–2012) of Collection 5 MOD13A standardised difference vegetation index (NDVI) data, with a spatial resolution of 30 arc-seconds (~1 km^2^). Finally, from 19 climate variables in the WorldClim database^41^, we selected variables that captured the main environmental gradients in the study area: mean annual temperature (BIO1) and annual precipitation (BIO12). WorldClim data were downloaded at a spatial resolution of 30 arc-seconds (~1 km^2^). The original spatial resolution of predictor variables was maintained to calculate the average of pixel values in each grid-cell by focal analysis. We then resampled the pixel size to 1 × 1 km to equal data scale for data processing. All spatial analysis were conducted using ArcGIS 10.4^42,43^. We analysed the pairwise correlation between explanatory variables: first removing those strongly correlated with coefficients of correlation above 0.80^44^ and then testing for multicollinearity in the data with the Variance Inflation Factor (VIF), by using stepwise elimination of highly inflating variables with a threshold of 10^23^. After these analyses, we removed slope, heat load index and distance to coast (Supporting Information, Appendix S1), resulting in eight variables selected for modelling: topographic ruggedness index, altitude, compound topographic index, annual maximum green vegetation fraction, annual mean temperature, annual precipitation, distance to cities and urban villages, and distance to rural villages.

**Table 1:** Description of the environmental predictor variables used to fit the species distribution models for Cuvier’s Gazelle.

Variables	Selected for modelling	Units	Source	Description	Calculation
***Topographic morphology***					
Altitude (ALT)	yes	meters	Elevation above sea level from SRTM (Shuttle Radar Topography Mission). 1 arc-second (~30 m^2^)	Elevation above sea level	
Slope (SLOPE)	no	degree		Terrain slope	Slope function. Toolbox. ArcGIS 10.4
Topographic Ruggedness Index (TRI)	yes	meters		^65^Topographic roughness. Calculated as the difference between the value of a cell and the mean of an 8-cell neighbourhood of surrounding cells	^66^Geomorphometric and Gradient Metrics Toolbox. ArcGIS 10.4
***Temperature-moisture regime***					
Heat Load Index (HLI)	no		Elevation above sea level from Shuttle Radar Topography Mission (SRTM). 1 arc-second (~30 m^2^)	^67^Potential direct incident radiation in relation with slope and aspect transformation	Geomorphometric and Gradient Metrics Toolbox. ArcGIS 10.4
Compound Topographic Index (CTI)	yes			^68,69^Flow accumulation by catchment size (wetness index) described as a function of both the slope and the upstream contributing area per unit width orthogonal to the flow direction and a quantification of catenary topographic convergence	
***Topographic distance and human factor***					
Distance to Coast (DISTCOAST)	no	meters	ArcGIS server	Terrain border	Euclidean distance. ArcGIS 10.4
Distance to Cities and Urban Villages (DISTSETT1)	yes		OpenStreetMap project and Haut Commissariat au Plan, Royaume du Maroc	Distance to the nearest human settlement with more than 1500 inhabitants	
Distance to Rural Villages (DISTSETT2)	yes			Distance to the nearest human settlement with less than 1500 inhabitants	
***Vegetation***					
Annual of Maximum Green Vegetation Fraction (AMGVF)	yes		USGS Land Cover Institute (LCI). 30 arc-seconds (~1km^2^)	Green vegetation fraction estimated from Normalized Difference Vegetation Index (NDVI)	Values calculated on 12 years (2001–2012) of Collection 5 MOD13A2 normalized difference vegetation index (NDVI)
***Climatic variation***					
Annual Mean Temperature (BIO1)	yes	degrees Celsius	WorldClim database 2.0, 30 arc seconds (~1km^2^)	Annual Mean Temperature	please merge with cell below
Annual Precipitation (BIO2)	yes	millimetres		Annual Precipitation	

### Species modelling strategy

The study area was enclosed in a 29 UTM (Universal Transverse Mercator) grid-cell measuring 5 × 5 km (Fig. 1). Due to the high mobility of gazelles, all records were gathered in each 5 × 5 km plot^45^ with a measure of occurrence (presence/absence). Presence was determined by dung middens and direct observations. To avoid imperfect detection in the absence of data (i.e. false negative surveys), we only included grids with transects of more than 5 km repeated at least twice^20^. Of the 84 surveyed grids, we removed those sites within a radius of 5 km to avoid spatial correlation, thus obtaining 51 grid-cells in all (41 positive cells and 10 negative cells). We then computed the nearest neighbouring index in ArcGIS 10.4 to prevent using clustered data^42^ (Supporting Information, Appendix S2).

**Figure 2:**
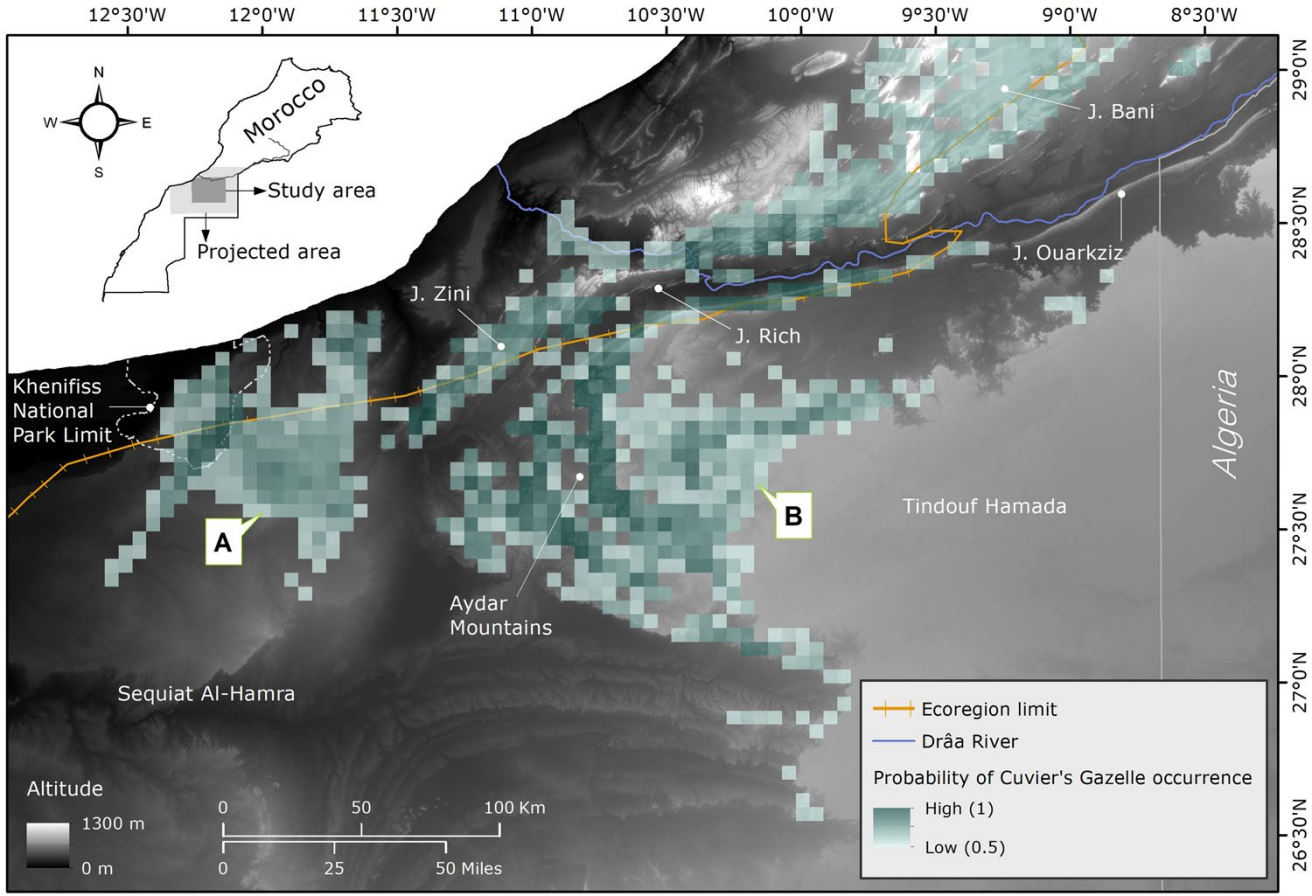
Spatial projection of the ensemble forecasting model to identify occurrence and suitable habitat of Cuvier’s Gazelle in the study area and surroundings. The probability of occurrence is ranked from low (0.5) to high (1) and shows two key areas “A” and “B” to consider in Cuvier´s Gazelle conservation plans. In dark orange the boundaries between ecoregions^70^ and in dark blue the Drâa River, considered as the northern limit of the Saharan population of the species. The forecast map also provides the main mountainous reliefs (Aydar Mountains, Jbel Zini, Jbel Rich, Jbel Ouarkziz and Jbel Bani). Basemap image by Shuttle Radar Topography Mission (SRTM)^38^. Software used: ArcGIS 10.4 (http://www.esri.com/)^39^ and R 3.6.2 (http://www.R-project.org/)^47^.

To build a species distribution model, we used the R package ‘biomod2’^46^ for R 3.6.2^47^. We used two algorithms common in distribution modelling: (1) Generalised Linear Model^48^ (GLM) and (2) Maximum entropy modelling of species distributions implemented in MAXENT 3.0.4 beta software^49^, to ultimately obtain an assembled model based on the variables we had selected. The use of an ensemble model or consensus algorithms in species distribution model is a powerful tool that prevents the selection of one single best model and thus eliminates or limits model selection bias while improving predictions of the current range of a species^23,50,51^. GLM is a flexible method and an extension of the linear regression models, which allows the response variable to follow a non-linear distribution and non-constant variance function. We fit GLMs for binary responses (the presence-absence data) using a logic link function, quadratic terms for each predictor and an automatic stepwise procedure with AIC. MAXENT is a reliable, effective technique based on a machine learning algorithm known as maximum entropy, which can use only presence, but also performs well when compared with presence-absence procedures that utilise both real and pseudo-absence data^52^. MAXENT was set up with 200 maximum iterations, with linear, quadratic, product, threshold and hinge features. We ran 10 sets of pseudo-absences (PA) and equalled the number of pseudo-absences as available presences to prevent a sampling bias^53^. To build a consensus ensemble forecasting model, we chose a mix of both algorithms that had shown good performance and therefore used them to project the potential spatial distribution in the region (Fig. 2).

### Model evaluation and spatial forecasting

The predictive power of occurrence in the models was tested using a split-sample cross-validation approach. To do this, the dataset was randomly split into two subsets: 75% for training data and 25% for testing data. This process was repeated 20 times for each model (× 10 pseudo-absence samplings × 2 algorithms). Therefore, 400 models were built in total. Models obtained through cross-validation were compared using a predictive accuracy metric: the area under the receiver operating characteristic curve (AUC). Only predictive models with a value of over 0.8 AUC were selected to create a final ensemble model. Differences in the predictive accuracies of different algorithms were tested using a Wilcoxon signed rank test. The models with a threshold that optimises AUC (above 0.8) were transformed into one single model by binary transformation, using the committee average approach in the training area^23,51,54^. In this process, the selected models are given equal probability levels by averaging the results of binary transformation and allowing direct comparisons across models^46,50^. To determine which environmental variables were most important, we selected 10 permutations to estimate variable importance in two ensemble methods: using algorithms and with all models. Then, the three ensemble model approaches (GLM, MAXENT and the consensus model) were analysed to assess the contribution and variable response of the predictive factors. To integrate results of different methods and avoid model selection bias^50,51^, we created a committed averaging map using the ensemble model of all algorithms to forecast the potential spatial occurrence in the southernmost known distribution area of Cuvier’s Gazelle. The threshold value below which the species was considered absent was <0.5^55^ with a range for probability of occurrence: low (0.5) to high (1). Then, the potentially suitable area was calculated by adding together all of the 5 × 5 km presence grid-cells, considering the species’ geographic Atlantic Saharan population border to be the Drâa River.

## Results

### Evaluation of models and habitat inferences

**Figure 3:**
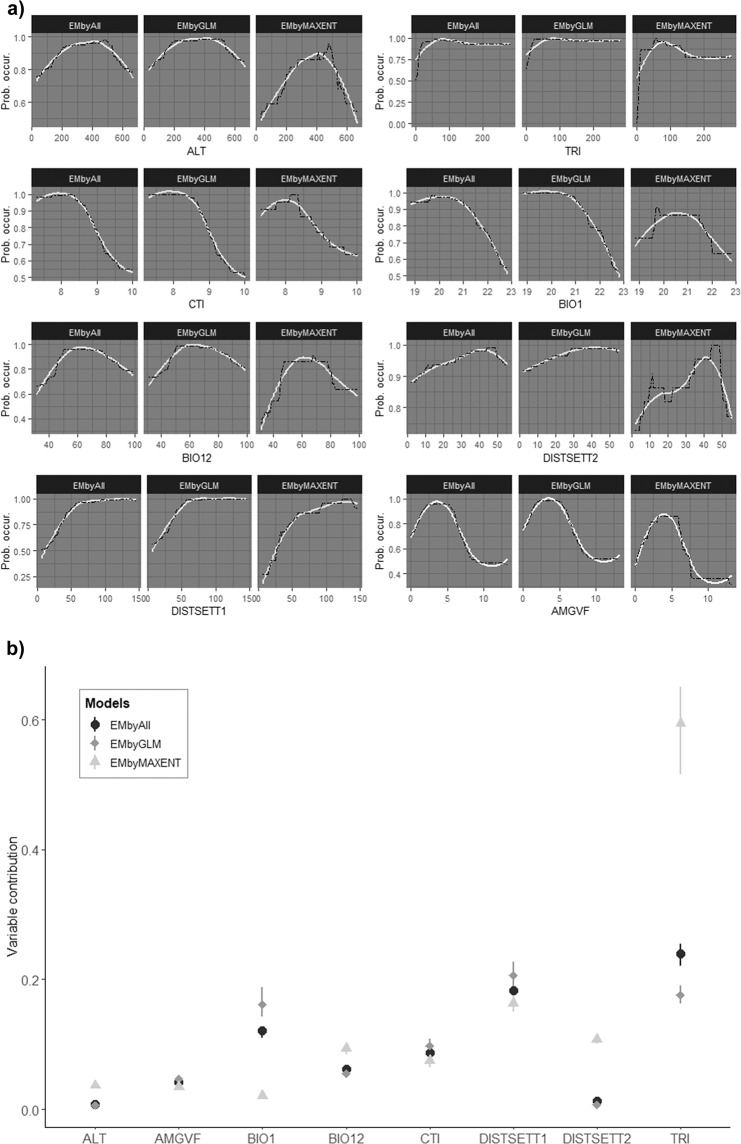
(**a**) Response curve and (**b**) variable contributions of the predictor variable selected for the different statistical approaches to model distribution of Cuvier´s Gazelle. Black curves in (**a**) are from original output data and grey curves by smooth processing. Models: ensemble model by GLM (EMbyGLM), ensemble model by MAXENT (EMbyMAXENT) and ensemble model by both algorithm (EMbyAll). Variables: altitude (ALT), annual of maximum green vegetation fraction (AMGVF), annual mean temperature (BIO1), annual precipitation (BIO12), compound topographic index (CTI), distance to cities and urban villages with >1500 inhabitants (DISTSETT1), distance to rural villages with <1500 inhabitants (DISTSETT2) and topographic ruggedness index (TRI).

Only 97 GLM and 43 MAXENT models with good accuracy (above 0.8 AUC) were selected to build three ensemble models. Significant differences were found between the two algorithms (W = 2682, p-value = 0.006), though GLM models performed better (AUC mean value: GLM, AUC = 0.86 SD = 0.048 and MAXENT, AUC = 0.83 SD = 0.039). However, the committee averaging ensemble model for MAXENT had the highest AUC value (Table 2). The eight variables selected for modelling showed different response curves, but the shape was similar for each variable in all ensemble models, except for distance to rural villages in MAXENT (Fig. 3a). The topographic ruggedness index and distance to cities and urban villages were key variables determining gazelle presence for the three ensemble models, though its respective contribution showed some differences (Fig. 3b): topographic ruggedness index was the most important variable for the consensus model and MAXENT, whereas distance to cities and urban villages was most important in GLM. In both cases, the probability of the Cuvier’s Gazelle occurrence increased with higher rugged terrain index and longer distances to cities and urban villages. Variables such as annual mean temperature in GLM and in the combined model, and distance to rural villages in MAXENT played a secondary role. In this instance, higher temperatures and a decrease in distance to rural villages affected negatively to gazelle presence. The rest of the variables in the different approaches showed low contribution determining gazelle presence (Fig. 3b).

**Table 2:** Predictive accuracy of ensemble models by binary transformation using the committee average approach in the training area for: a) GLM (EMbyGLM), b) MAXENT (EMbyMAXENT) and c) both algorithms (EMbyAll).

Type of ensemble model	AUC	Sensitivity	Specificity
a) EMbyGLM	0.86	92.68	69.28
b) EMbyMAXENT	0.90	87.81	77.74
c) EMbyAll	0.88	95.12	67.40

### Predicted distribution range and priority conservation areas

Considering the northern limit of our study area to be the Drâa River, the predicted suitable Cuvier’s Gazelle habitat resulting from the consensus ensemble forecasting model yielded an area of 15820 km^2^ (Fig. 2). The forecasted potential spatial occurrence at its southernmost limit of distribution revealed two well-demarcated areas: “A” and “B,” with estimated areas of 4385 km^2^ and 11315 km^2^, respectively (Fig. 2). These areas are regions lacking formal protection, with only a relatively low percentage (5%) included in Khnifiss National Park. Presence of Cuvier’s gazelles was higher in the eastern area of Khnifiss National Park, on the steep slopes of the Hammada of Tindouf, in the mountainous terrain of Aydar and across the large reliefs in the area (Jbels Zini, Rich and Ouarkziz). The consensus ensemble forecasting model showed a continuous suitable habitat strip to the north of the Drâa River, in the Jbel Bani and beyond the limits of the ecoregion.

## Discussion

Our study illustrates an example of how to successfully deal with a lack of key data in developing well-designed wildlife conservation actions in remote areas, such as the hard environment of the Sahara Desert, through combining large scale field surveys and distribution modelling. This approach has clear applications for any type of study on the population distribution and dynamics of threatened species that live in these types of regions, not only wild ungulates but also carnivores or other mammals of medium to large size. The present study provides robust ensemble models that achieved a good estimate of the importance of the variables and their response curves^23,46,56^. Our analyses confirm that the species presence and distribution of Cuvier’s Gazelle in the study area are primarily influenced by the variability or complexity of the terrain and the increasing distance to large human settlements. As a result, we found a higher projected probability of Cuvier’s Gazelle presence in zones with a complex network of hills and ravines and in remote areas with a low human population density and less accessibility (e.g. paved roads). These results are consistent since Cuvier’s Gazelle is well-adapted to rugged mountain areas^57^, where they can find shelter from predation and poaching, but also where environment conditions are more favourable against the extreme Saharan climate. In such areas, Cuvier’s Gazelle can feed in ravines and dry river basins, where vegetation remains productive throughout the year. However, we were able to confirm that they avoid the more productive *oueds*, which are located in flat areas, probably because of greater pressure from livestock and human presence, factors that may hold great importance in habitat selection in the area^20^. This is consistent with the annual maximum green vegetation fraction and the compound topographic response curves, both positively correlated to the size of river basins, and therefore more productive *oueds*. The human factor represented by the distance to the nearest large human settlement is one of the many factors constraining the potential distribution of the species. A similar phenomenon was described in Chammem *et al*.^35^, in which the species presence and distribution for dorcas gazelle in Tunisia seemed to be affected negatively by human presence and land use, rather than habitat characteristics. The projected species distribution model area probably reflects a shelter range that allows survival in apparently suboptimal conditions, due to both poaching and the extreme Saharan environment, where the species reaches the southernmost limit of its range. The impact of illegal wildlife hunting is one of the causes or the main cause of decline and extinction of ungulate species throughout the Sahara Desert^7,8,10,16,58^, as it is in Morocco^59,60^. Therefore, the better-preserved Cuvier’s Gazelle populations in Morocco may currently be related to remote territories that have a low human population density. Furthermore, the species shows high environmental heterogeneity throughout its range in North Africa^18^. Cuvier’s Gazelle and slender-horned gazelle were suggested to inhabit two different ecotypes of mountain and lowland sand dunes, respectively, with a parapatric population structure^17^, which may explain the ecological flexibility of Cuvier’s Gazelle and thus its survival under extreme environmental conditions. In the study area, other threats such as overgrazing and habitat loss must be studied, but at least do not seem to have had major effects thus far, in principle. Nevertheless, this situation appears to be changing. Recently, we noticed an increase in the number of domestic grazers and local pastoralists. This appears to be related to the opening of new roadways (some paved) and the use of large flexible plastics for water storage, allowing for more stable livestock exploitation in areas far from water sources.

### Implications for conservation policies

At present in Morocco, two main populations of Cuvier’s Gazelle have been identified: the Bas Drâa-Aydar region (our study area) and the western Anti-Atlas^57^. Moreover, small populations are located in the Western High Atlas (north of Agadir) and on the south side of the central High Atlas and East Atlas^60^. We extended the species distribution model forecast area to the south of its known distribution area, identifying two well-delimited key areas for Cuvier’s Gazelle (A-B; Fig. 2), a result consistent with the previous scattered and opportunistic records inside and outside of the study area, which resemble our species distribution model^9,57,60^. Within both areas, our species distribution model identified sectors with high Cuvier’s Gazelle probability of presence (close to 1). These locations hold a suitable habitat where action aimed at conservation is urgent. The whole region is considered public land under the Moroccan government’s management, with nomads moving about in temporary camps with herds of goats, sheep and dromedaries. During the field surveys, we observed that these people opportunistically try to hunt gazelles, whereas groups of poachers coming from the nearest large cities operate at will. Moroccan authorities planned to declare one national park (Bas Drâa NP), one Biological and Ecological Interest Site (Oued Tirhzer) as well as two hunting reserves (Messeied-Abeteih and Oued Chbeyka)^61,62^. However, the surface area covered by these planned protected areas is insufficient to protect a key space for Cuvier’s Gazelle (i.e. a viable population), since it would cover just 25% of the range we have estimated and, moreover, these projects have been stalled for more than two decades. Moreover, local Cuvier’s Gazelle densities are quite low (0.08 individuals/km^2^) and the total population has been estimated at 935 (95% CI 597–1607) individuals^20^. Our results show the need to extend the protected areas designed in the Moroccan strategy for the preservation of endangered species^60^, as well as the urgency in the implementation of this national strategy to protect the current natural populations of Cuvier’s Gazelle. New protection areas forming a backbone for the region’s sustainable development with proper protection (in terms of poaching) and a low human impact will be crucial to preserving the species. The Cuvier’s Gazelle population inhabiting this region will have a better chance against any harsh conditions (water shortages and extremely high temperatures) in a changing climate scenario^7^. Furthermore, the region has been proposed as a dispersal corridor and fauna shelter for the Sahara-Sahel region^16^. These features make this part of Morocco a crucial area not only for the Cuvier’s Gazelle preservation, but also for other endangered ungulate species still present in the region such as dorcas gazelle and Saharan barbary sheep (*Ammontragus lervia sahariensis*), and carnivores such as striped hyaena (*Hyaena hyaena*), sand cat (*Felis margarita*), caracal (*Caracal caracal*) and, in all likelihood, the critically endangered Saharan cheetah (*Acinonyx jubatus hecky*). The latter could find a strategic area for recovery here, the area could also aid in the recovery of other extinct ungulate species like mhorr dama gazelle (*Nanger dama mhorr*), scimitar-horned oryx and addax, through reintroduction projects. Some of these species have begun to be reintroduced in the Souss-Massa region^63,64^. The current work can provide a basis for the definitive design of protected areas and reintroduction projects.

## Supplementary information

Appendix S1 and S2.

## Data Availability

The datasets generated and/or analysed during the current study are available from the corresponding authors upon reasonable request.
