# Publisher Correction: Identifying priority conservation areas in a Saharan environment by highlighting the endangered Cuvier’s Gazelle as a flagship species

**DOI:** 10.1038/s41598-020-69588-6

**Published:** 2020-07-29

**Authors:** F. Javier Herrera-Sánchez, Jose María Gil-Sánchez, Begoña Álvarez, Inmaculada Cancio, Jesus de Lucas, Ángel Arredondo, Miguel Ángel Díaz-Portero, Javier Rodríguez-Siles, Juan Manuel Sáez, Joaquín Pérez, Emil McCain, Abdeljebbar Qninba, Teresa Abáigar

**Affiliations:** 1 Harmusch, Study and Conservation of Wildlife, C/San Antón 15, 1°, E 13580, Almodóvar del Campo, Ciudad Real Spain; 2 Département de Zoologie et Ecologie Animale, Institut Scientifique de Rabat, Université Mohammed, V. Av. Ibn Battouta, BP 703, 10090 Agdal, Rabat, Morocco; 3 Estación Experimental de Zonas Áridas (CSIC), Crta. Sacramento s/n, 04120 La Cañada de S. Urbano, Almería Spain

Correction to: *Scientific Reports* 10.1038/s41598-020-65188-6, published online 19 May 2020

As a result of a production error, the version of Table 1 originally published with this Article contained a typesetting comment. This comment has now been removed in the PDF and HTML versions of the paper.

