# Supplementary material for: Identifying priority conservation areas in a Saharan environment by highlighting the endangered Cuvier’s Gazelle as a flagship species
Source: Sci Rep. 2020 May 19;10:8241. doi: 10.1038/s41598-020-65188-6 (PMC7237411; doi:10.1038/s41598-020-65188-6)
Supplement: Supplementary file 1 — Appendix S1 and S2. [file 41598_2020_65188_MOESM1_ESM.pdf]

Identifying priority conservation areas in a Saharan environment by highlighting the endangered Cuvier's Gazelle as a flagship species

F. Javier Herrera-Sánchez, Jose María Gil-Sánchez, Begoña Álvarez, Inmaculada Cancio, Jesus de Lucas, Ángel Arredondo, Miguel Ángel Díaz-Portero, Javier Rodríguez-Siles, Juan Manuel Sáez, Joaquín Pérez, Emil McCain, Abdeljebbar Qninba & Teresa Abáigar

SUPPLEMENTARY INFORMATION

Appendix S1. Matrix of correlation coefficients and Variance Inflation Factor (VIF) analysis[1]. Pre-selection of variable by removing environment metrics highly correlated and with multicollinearity problems. Variables removed in "red".

- Matrix correlation table

	Alt	*Slope*	*Tri*	*Hli*	*Cti*	*Amgvf*	*BIO1*	*BIO12*	*Distcoast*	*Distcities*	*Distvillages*
Alt	1.00										
Slope	0.04	1.00									
Tri	0.06	0.96	1.00								
Hli	0.03	0.89	0.86	1.00							
Cti	0.10	-0.85	0.73	0.73	1.00						
Amgvf	0.42	0.19	0.19	0.14	0.22	1.00					
BIO1	0.17	-0.37	0.35	0.32	0.30	-0.39	1.00				
BIO12	0.03	0.50	0.49	0.44	0.38	0.33	-0.80	1.00			
Distcoast	0.63	-0.35	0.31	0.31	0.23	0.03	0.62	-0.69	1.00		
Distcities	0.40	-0.28	0.31	0.24	0.15	0.04	0.45	-0.70	0.63	1.00	
Distvillages	0.26	-0.24	0.24	0.24	0.19	0.00	0.01	-0.28	0.27	0.21	1.00

- Variance Inflation Analysis.

a) 1 variables (Distcoast) from the 9 input variables had collinearity problem.

b) After excluding the collinear variables, the linear correlation coefficients ranges between

min correlation (distvillages ~ amgvf): 0.0029392

max correlation (BIO12 ~ BIO11): -0.7952318

-------- VIFs of the remained variables --------

	VARIABLES	VIF
1	Alt	1.685919
2	Tri	2.494449
3	Cti	2.268704
4	Amgvf	1.478186
5	BIO1	3.483616
6	BIO12	6.110327
7	Distcities	2.91219
8	Distvillages	1.349295

Reference:

1. Hair, J.F., Black, W.C., Babin, B.J., Anderson, R.E. *Multivariate Data Analysis, 6th edn* (Pearson Prentice Hall, 2006).

Appendix S2. Nearest Neighbour Index (NNI) from 51 surveyed grids in the sampling area. Given the z-score of 0.2044, the pattern does not appear to be significantly different than random. NNI is based on the average distance from each feature to its nearest neighbouring feature and it was implemented through the "Spatial Analyst" extension of ArcGIS 10.4 (ESRI Inc., http://www.esri.com/).).

----Average Nearest Neighbour Summary----

Nearest Neighbour Ratio: 1.014967
z-score: 0.204487
p-value: 0.837973

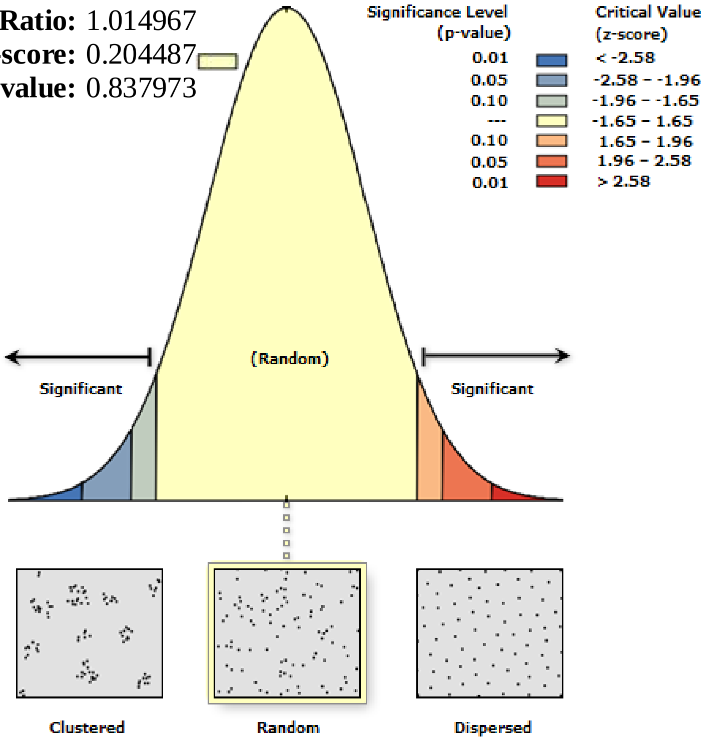

Average Nearest Neighbour Summary

**Observed Mean Distance:**	13568.4681 Meters
**Expected Mean Distance:**	13368.3771 Meters
**Nearest Neighbour Ratio:**	1.014967
**z-score:**	0.204487
**p-value:**	0.837973
